# Determining habitat quality for species that demonstrate dynamic habitat selection

**DOI:** 10.1002/ece3.1813

**Published:** 2015-11-19

**Authors:** James M. Beerens, Peter C. Frederick, Erik G. Noonburg, Dale E. Gawlik

**Affiliations:** ^1^ Wetland and Aquatic Research CenterU.S. Geological SurveyFort LauderdaleFlorida; ^2^ Department of Wildlife Ecology and ConservationUniversity of FloridaGainesvilleFlorida; ^3^ Department of Biological SciencesFlorida Atlantic UniversityDavieFlorida; ^4^ Department of Biological SciencesFlorida Atlantic UniversityBoca RatonFlorida

**Keywords:** Environmental gradients, habitat quality, habitat selection, hydrology, prey availability, restoration, species distribution model, wading birds

## Abstract

Determining habitat quality for wildlife populations requires relating a species' habitat to its survival and reproduction. Within a season, species occurrence and density can be disconnected from measures of habitat quality when resources are highly seasonal, unpredictable over time, and patchy. Here we establish an explicit link among dynamic selection of changing resources, spatio‐temporal species distributions, and fitness for predictive abundance and occurrence models that are used for short‐term water management and long‐term restoration planning. We used the wading bird distribution and evaluation models (WADEM) that estimate (1) daily changes in selection across resource gradients, (2) landscape abundance of flocks and individuals, (3) conspecific foraging aggregation, and (4) resource unit occurrence (at fixed 400 m cells) to quantify habitat quality and its consequences on reproduction for wetland indicator species. We linked maximum annual numbers of nests detected across the study area and nesting success of Great Egrets (*Ardea alba*), White Ibises (*Eudocimus albus*), and Wood Storks (*Mycteria americana)* over a 20‐year period to estimated daily dynamics of food resources produced by WADEM over a 7490 km^2^ area. For all species, increases in predicted species abundance in March and high abundance in April were strongly linked to breeding responses. Great Egret nesting effort and success were higher when birds also showed greater conspecific foraging aggregation. *Synthesis and applications:* This study provides the first empirical evidence that dynamic habitat selection processes and distributions of wading birds over environmental gradients are linked with reproductive measures over periods of decades. Further, predictor variables at a variety of temporal (daily‐multiannual) resolutions and spatial (400 m to regional) scales effectively explained variation in ecological processes that change habitat quality. The process used here allows managers to develop short‐ and long‐term conservation strategies that (1) consider flexible behavioral patterns and (2) are robust to environmental variation over time.

## Introduction

Understanding how species are linked with their habitat, such as determining what resources and conditions are necessary for occupancy, survival, and reproduction, is integral to effectively managing wildlife populations (Morrison et al. 2012). Predictability of habitat occupancy, termed habitat evaluation (Van Horne 1983), is highest when the underlying mechanisms that relate habitat to survival and reproduction are known (Bock and Jones 2004). The complex process of habitat evaluation involves determining how patterns of habitat use in proportion to habitat availability indicate habitat selection, how selection reflects preference, and how preference is shaped by differential fitness in response to heterogeneity of habitat resources (Garshelis 2000, Lele et al. 2013).

In most habitat selection studies, it is assumed that there is a direct linkage among habitat selection, abundance or occurrence, and habitat quality, which affects fitness (Bock and Jones 2004, Boyce et al. 2015). On the basis of that assumption, ecologists typically proceed to identify environmental variables that are selected or avoided and consider these to be important for population persistence (Aldridge and Boyce 2007). Environmental variables may represent food availability, shelter from predators, or distance from negative anthropogenic effects. However, if animals are unable to directly assess habitat quality and instead use environmental cues such as the presence of conspecifics, habitat selection can reduce fitness (i.e., ecological or perceptual traps; Patten and Kelly 2010, Gilroy et al. 2011, Hollander et al. 2011). In these cases, habitat selection models can be misleading (Gaillard et al. 2010) or have limited predictive power (Folmer and Piersma 2012). Many studies report selection and preference based on a use versus availability framework (Manly et al. 2002), but few have linked selection of specific resources to fitness (Whitham 1980, Hollander et al. 2011, Stephens et al. 2015), especially in dynamic environments where resources change daily. Thus, there is much impetus to establish a direct relationship by connecting shifting patterns of selection and spatio‐temporal distributions to measures of fitness (Mosser et al. 2009, Nielsen et al. 2010).

While experiments can reveal preferences by controlling resource availability and measuring resource use by a species, preference in natural settings must be inferred from patterns of observed use in environments while accounting for changing resource levels. Some habitat selection models determine habitat preference even with changing habitat conditions (Arthur et al. 1996; functional response in habitat selection; Mysterud and Ims 1998). This allows plasticity in foraging behavior to be incorporated into models of systems with variation in the resource base (Gillies et al. 2006, Hebblewhite and Merrill 2008, Godvik et al. 2009).

Relationships between habitat and species occurrence or abundance may also be nuanced as a result of ecological processes operating at different spatial and temporal scales (Boyce et al. 2015). Species occurrence and density can be disconnected from static measures of habitat quality when resources are highly seasonal, unpredictable over time, and patchy (Van Horne 1983). When spatial and temporal variation in resources occurs, scale‐specific heterogeneity of the environment can often drive the selection response and produce despotic distributions apparently mismatched to habitat quality (Calsbeek and Sinervo 2002, Gaillard et al. 2010). Furthermore, habitat selection is hierarchical and individuals use locations in response to limiting processes at multiple spatial (or temporal) scales (Johnson 1980). Individuals might employ different selection responses at different scales, so as to maximize fitness given the set of resources available (Resetarits 2005, Godvik et al. 2009). For example, within‐year habitat selection that correlates positively with survival in the short term could come at the expense of reproductive success in the long term (McLoughlin et al. 2007). The models resulting from such a short‐term study would imply that the system be managed to increase the short‐term process, potentially resulting in lower fecundity (Aldridge and Boyce 2007). Thus, long‐term studies of habitat selection linked with abundance and measures of fitness are necessary to understand the differing selection strategies and species distributions across resource gradients.

**Figure 1:**
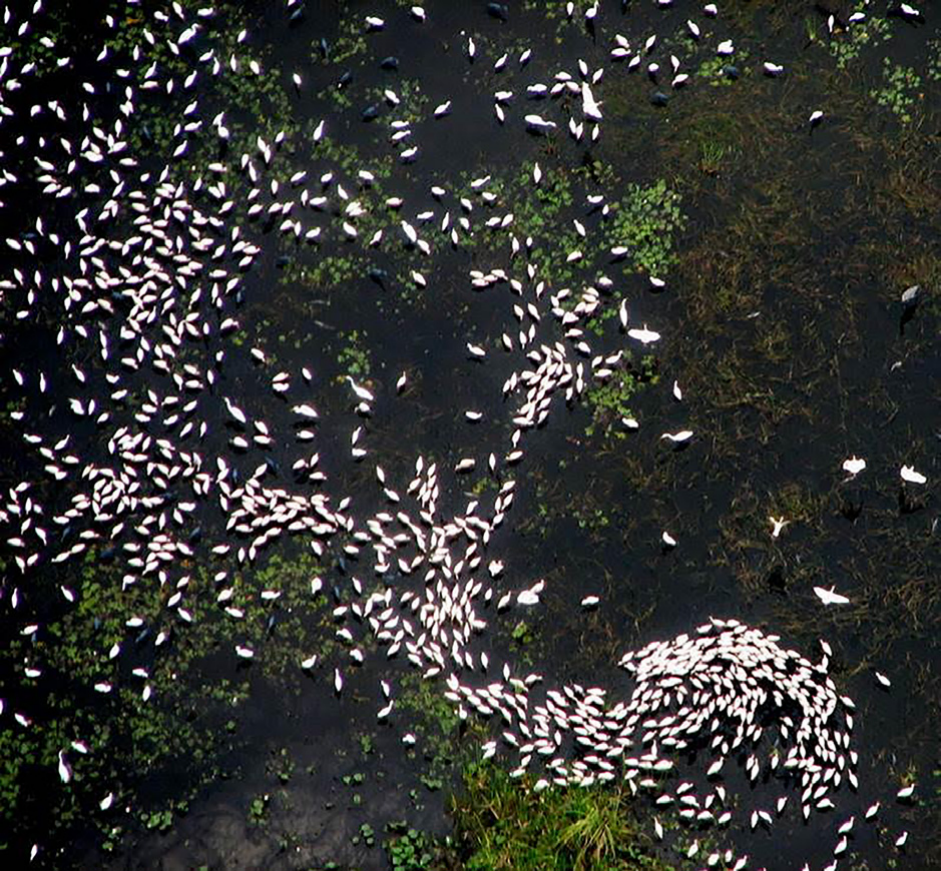
Mixed species foraging distribution of wading birds. Length of inundation over periods of months and years increases the production of the aquatic prey base and dry season drying and ponding of water over periods of weeks concentrates prey and is linked to wading bird foraging density. Photograph by James M. Beerens.

In the Everglades of Florida, varying resource selection strategies across temporal scales are exemplified by several species of long‐legged wading birds (White Ibis [*Eudocimus albus*], Great Egret [*Ardea alba*]; Beerens et al. 2011), whose breeding populations are limited by food availability (Frederick and Spalding 1994, Ogden 2005, Herring et al. 2010), and availability of prey is driven by water depth and density of prey (Gawlik 2002). In this shallow, subtropical wetland system, length of inundation over periods of months and years increases the production of the aquatic prey base (Trexler 2010), whereas dry season drying and ponding of water over periods of weeks concentrates prey and is linked to wading bird foraging density (Russell et al. 2002; Fig. 1). Great Egrets and White Ibises (hereafter egret and ibis) selected foraging sites strongly influenced by dry season ponding (short term) in a year with reduced prey availability and limited nesting success, but length of inundation (long term) was more influential in a year with greater prey availability (Beerens et al. 2011). This demonstrates that birds are affected by a hierarchy of temporally nested processes at the landscape scale. Understanding the interaction between these processes and how it impacts species occurrences and abundances requires a dataset that includes varying combinations of resource levels over multiple years. This understanding is significant in the Everglades because management recommendations from habitat selection studies guide long‐term (30 year) and large‐scale (cf 4000 km^2^) restoration projects.

In the current study, we examined the association between resource availability, habitat selection, abundance, and occurrence predictions using a multimodel wading bird distribution and evaluation (WADEM; Beerens et al. 2015) framework and used this model to predict metrics of reproductive effort and success. Using distribution and hydrologic data, WADEM estimates daily changes in (1) selection across resource gradients, (2) landscape abundance of flocks and individuals, (3) conspecific foraging aggregation, and (4) resource unit occurrence (at fixed 400 m cells) in a rapidly changing environment. We predicted that the strong interactions among resources linked to differing temporal scales, such as the combined requirement for high prey production (multiyear hydrologic processes) and high prey density (within‐year drying processes), and selection responses to these resources, would drive variation in both nesting effort and success. Further, we expected that the timing of peaks in the outputs of WADEM during key periods of the breeding cycle would be linked to responses at different phases of breeding. We predicted that dispersion of foraging individuals would be an important consideration in understanding breeding, because high patch quality might result in strong clustering of individuals in preferred habitat, whereas more uniform low patch quality might result in a smaller ratio of individuals: flocks. We expected this ratio to vary among species as foraging conditions change during the dry season and for this clustering to be positively related to habitat quality for species with higher foraging interference costs.

## Materials and Methods

### Study area

The Florida Everglades is a dynamic subtropical wetland subject to rapid seasonal spatial and temporal resource pulses (Frederick et al. 2009). Wading birds time their breeding to coincide with these resource pulses; however, their populations have declined by an estimated 70% since the 1930s (Crozier and Gawlik 2003) because of habitat reduction and hydrologic alteration. Thus, a goal of ongoing management and restoration of the Everglades is to provide ecological functions more similar to the historical system, signified by various wading bird nesting patterns (Frederick et al. 2009).

### Wading bird distribution and evaluation models

The WADEM framework consists of two components, the temporal foraging conditions (TFC) and spatial foraging conditions (SFC) models (Beerens et al. 2015). These models were parameterized by pairing Systematic Reconnaissance Flight (SRF) occurrence data for Great Egrets, White Ibises, and Wood Storks (*Mycteria americana*; hereafter stork) from 2000 to 2009 with hydrologic variables derived from the Everglades Depth Estimation Network (EDEN). SRFs have been consistently used from 1985 to 2012 to document the abundance, flock composition, and spatiotemporal distribution of foraging wading birds across the Greater Everglades system (Water Conservation Areas [WCA], Big Cypress National Park [BCNP], and Everglades National Park [ENP]). Each year from January to June, low altitude (61 m) aerial surveys were used to estimate numbers and species of birds in belt transects spaced at 2 km intervals (Bancroft et al. 1994). The EDEN is an integrated 400 m grid network of real‐time water‐level monitoring, ground elevation data, and surface water modeling that provides estimated water depth information for the entire freshwater portion of the greater Everglades (Telis 2006).

**Figure 2:**
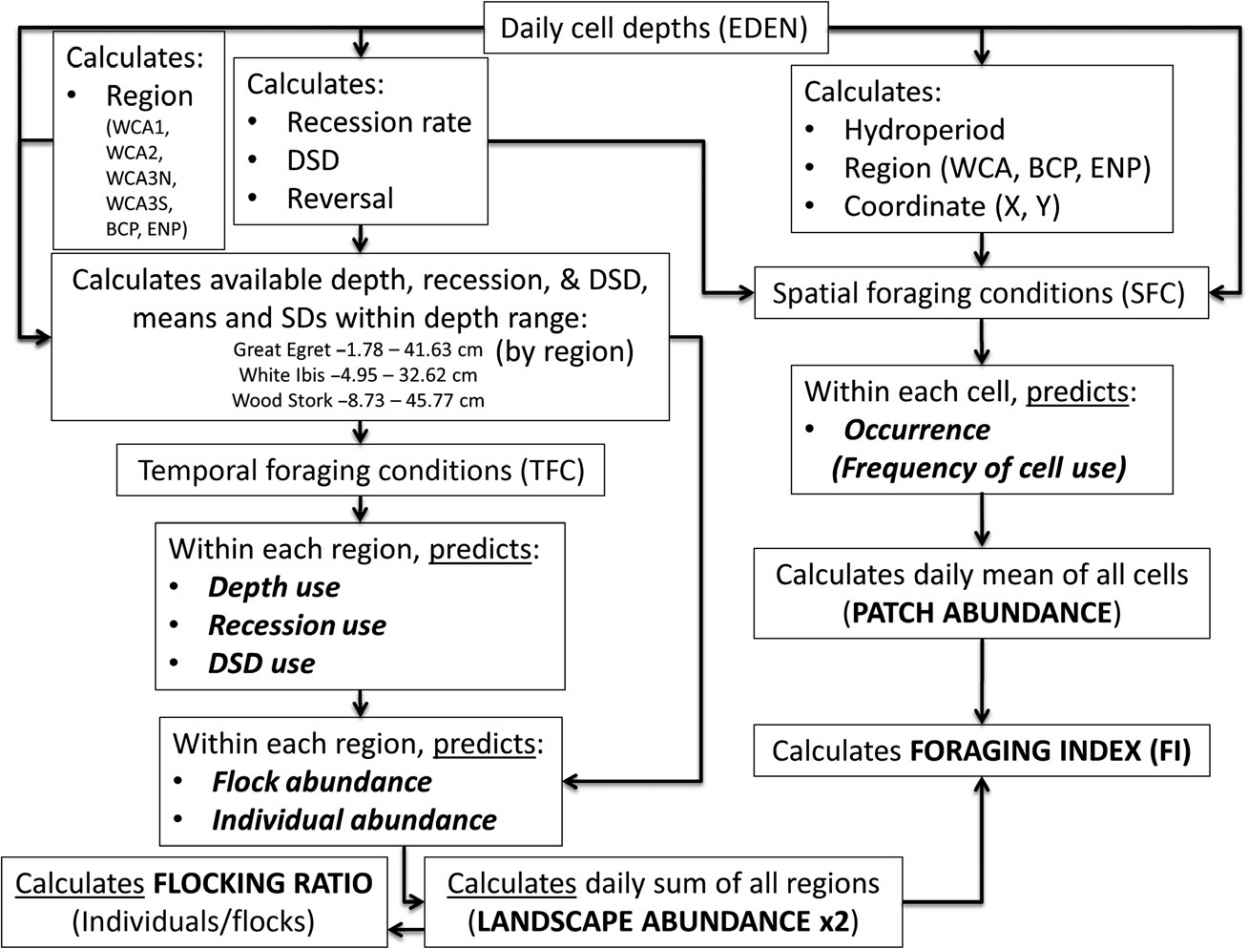
Conceptual model flow of the wading bird distribution and evaluation models (WADEM). Hydrologic variables were calculated using water depths from the Everglades Depth Estimation Network (EDEN) to estimate resource availability occurring over differing time scales. In the temporal foraging conditions models (TFC), resource availability is used to predict resource use (i.e., selection), which is then used to predict the abundance of flocks and individuals across the study area. In the spatial foraging condition models (SFC), average resource use is used to predict wading bird occurrence in a cell over the study period. These WADEM outputs are italicized in bold and are summarized to serve as model inputs for this study (capitalized in bold) to predict species‐specific nesting effort and success.

**Figure 3:**
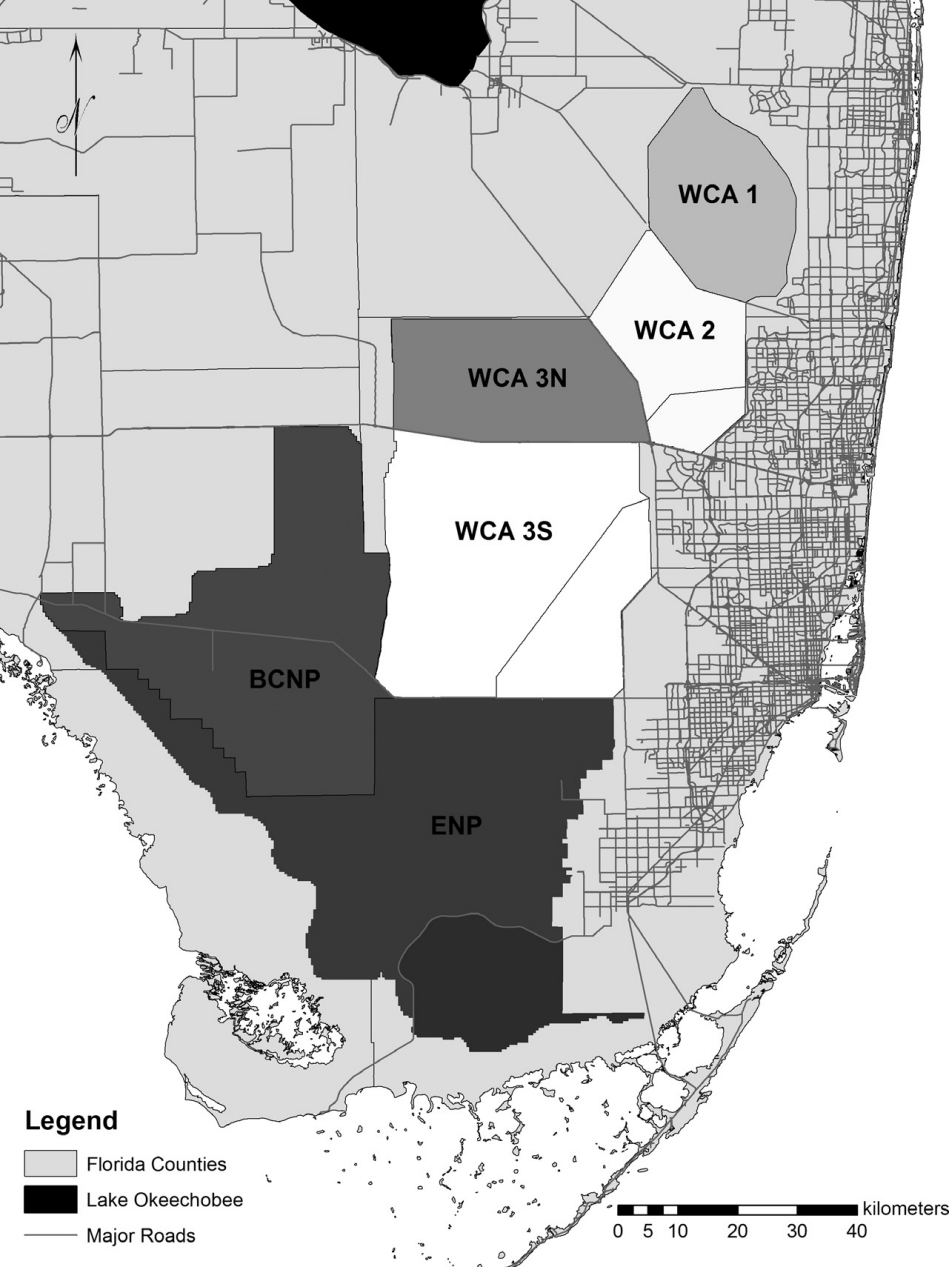
South Florida study area displaying Everglades hydrologic basins (regions). The regions of coverage include Water Conservation Areas (WCA) 1, 2, 3‐North, 3‐South, Big Cypress National Park (BCNP), and Everglades National Park (ENP).

The TFC models used three hydrologic variables calculated across the EDEN domain as proxies for prey dynamics (Fig. 2). These variables represent hydrologic conditions across a gradient of temporal scales: (1) days since drydown (DSD) was used as an indicator for long‐term prey production and immigration into an EDEN cell (DeAngelis et al. 2005, Trexler 2010), (2) recession rate was used as an indicator for prey concentration dynamics at an intermediate scale (Russell et al. 2002, Beerens et al. 2011), and (3) daily water depth was used as an indicator of short‐term prey availability (Gawlik 2002, Beerens et al. 2011). These indicators, which we refer to as “resources”, were considered available only when cell depths were in the foraging depth range of each species (determined from distribution data; Beerens et al. 2015; Fig. 2). For each day an SRF was conducted, we calculated the mean and standard deviation (SD) of the three resources over the EDEN cells that were surveyed (within the available habitat of each region). The means, their interactions, and SDs were used to predict daily use of the three resources, which subsequently was used to predict the daily abundance of flocks and individuals for each target species across the landscape. Flock presence was defined for each target species as one or more birds of that species detected in a cell, whereas individual abundance was the total number of birds present. Both individual and flock responses were modeled because wading birds are highly social and select foraging habitat based in part on the presence of conspecifics, a process that may increase or decrease individual fitness (Campomizzi et al. 2008). For the TFC, daily output summed over each region (WCA‐1, WCA‐2, WCA‐3N, WCA‐3S, BCNP, and ENP; Fig. 3) represents predicted SRF abundance of flocks and individuals in the landscape (Beerens et al. 2015; Fig. 2).

The SFC models used a different approach by (1) using cumulative foraging observations in each EDEN cell to predict frequency of use of a cell from hydrologic conditions at that location and (2) assessing spatial correlation in hydrologic variables to account for topographic patterns that span multiple cells (Beerens et al. 2015). By accounting for patterns in the spatial variation of the landscape, the noise independent of the hydrologic parameters can be reduced to better capture the species‐specific behavioral response to rapidly changing habitat conditions (Dormann 2007). In addition to depth, recession, and DSD, the SFC used dry‐to‐wet reversal and hydroperiod to capture additional hydrologic processes (Fig. 2). The dry‐to‐wet reversal variable estimated the timing of when a cell had gone dry and rewet (within a dry season), which results in highly depleted fish populations (Trexler et al. 2002). Hydroperiod was defined as the 10‐year mean annual length of cell inundation, which influences wading bird distributions through long‐term changes in microtopography and vegetation communities (Gunderson 1994). The hydrologic variables, averaged over each observation, and the interactions of the three resources predicted frequency of bird occurrence (defined as the number of times over the study period that a species was detected in a given 400‐m cell) with the expectation that hydrologic variables would converge on optimal values the more a cell was frequented. Output from the SFC averaged over the landscape can serve as a surrogate measure of the abundance of high‐quality patches (defined here as patch abundance; Fig. 2).

### Nesting effort

Egret, ibis, and stork maximum nesting effort (numbers of nest starts – presence of an incubating parent) from 1993 to 2013 was obtained from annual South Florida Wading Bird Reports (South Florida Water Management District, West Palm Beach, FL) and Crozier and Gawlik (2003). Nesting effort was monitored annually throughout the study area by systematic aerial colony search and survey efforts (February–June, monthly) performed at 240 m altitude by two observers in overlapping east–west transects, through targeted ground visits to colonies, and through systematic ground surveys for small colonies by airboat (see Frederick and Ogden 2003 for further details of the monitoring program).

### Nesting success

Nesting success was monitored by checking individually identifiable nests every 5–7 days during 1993–2013. Nesting success was defined as having at least one young reach the ages of 14 or 21 days posthatching. The cutoff points were determined as the age at which researchers could reliably associate individual young with their nest. Beyond these ages, young leave the nest but stay nearby and receive food from parents for 2–5 weeks. White Ibises and herons in the genus *Egretta* develop more quickly and were considered fledged at 14 days posthatching (see McVaugh 1972). For larger and more slowly developing Great Egrets, the age was 21 days posthatching. Nesting success for storks was not determined because of small sample sizes during the EDEN period of record. Colonies monitored in each year were selected based on size (largest 3–5 colonies), species composition, and geographic representation. Many colonies are occupied by several species, and not all colony locations are active in each year (Frederick and Spalding 1994). In monitored colonies, all nests were within belt transects 4 m in width and oriented from the colony edge to areas of greatest nest density and were marked with numbered flagging. Colonies were monitored from the time most nests had progressed to incubation until all nests on transects had either failed or fledged young. On each visit, all nests were checked for contents. Nests were identified to species based on construction materials, size, and egg and chick characteristics (McVaugh 1972). Nest start date was defined as the date of laying of the first egg, determined based on either laying or hatching schedule. Nests were assumed to have failed when all eggs or chicks disappeared or were found dead prior to the fledging age. Barring more detailed evidence at the nest, timing of nest failure was assigned to the midpoint between nest checks (Manolis et al. 2000). Nesting success was then expressed as daily nest success probabilities for each species, summed over the number of exposure days, and averaged over all colonies within a breeding year using the methods in Mayfield (1961, 1975) and Hensler (1985).

### Variables and statistical methods

We applied the WADEM to hydrologic data calculated from the EDEN during the dry seasons (January–May) and species‐specific nesting effort and nesting success (ibis and egrets only) estimates from field surveys 1993 to 2013. The response variable, stork nesting effort, was fourth‐root transformed to meet the assumption of normality, whereas egret and ibis nesting effort did not require transformations to meet the assumptions. The daily foraging index (FI) was calculated by multiplying daily individual abundance (TFC) by daily patch abundance (SFC) to jointly account for the seasonal increase and eventual decrease in patch abundance as the landscape dries, and the increase in species abundance as habitat with longer periods of inundation becomes available in suitable water depths (Beerens et al. 2015; Fig. 2). A predicted flocking ratio was obtained by dividing individual abundance by flock abundance to determine the mean number of individuals per flock for a target species on a given day (Fig. 2). The flocking ratio was then averaged over the dry season to indicate the annual degree of foraging aggregation (*Ratio*) and its variation (*Ratio SE*). For all analyses, the standard error was chosen over the SD to represent variability in the indices based on higher model support (Burnham and Anderson 2002). The daily change in individual abundance, calculated to determine whether predicted birds were increasing or decreasing, was averaged by month to indicate monthly changes in foraging conditions (*Jan* Δ, *Feb* Δ, *Mar* Δ, and *Apr* Δ). These changes were included in the model set to focus on the time frame when adults and nestlings were more sensitive to fluctuations in habitat quality. In addition, the daily individual abundances and FI were averaged by month (*Jan* $\bar{x}$, *Feb* $\bar{x}$, *Mar* $\bar{x}$, *and Apr* $\bar{x}$) to determine whether the timing of the absolute level in individual abundance or FI was driving nesting effort and success. The mean dry season flock and individual abundance, FI, and their variations (SE) were also tested to determine whether overall annual patterns explained additional variation in nesting effort and success.

**Figure 4:**
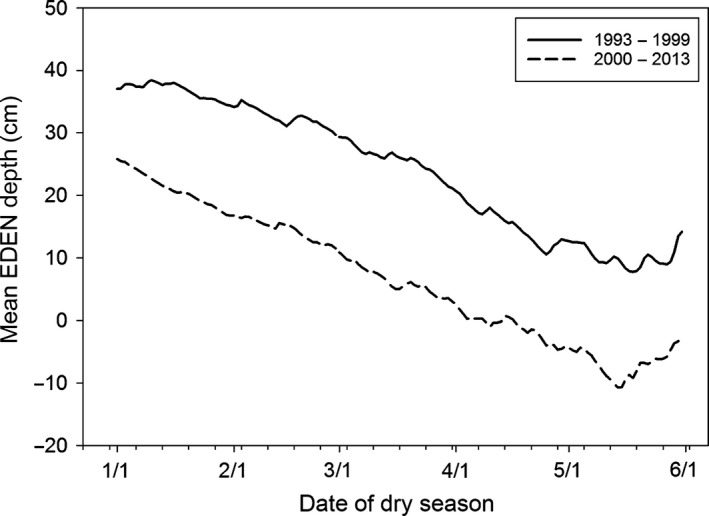
Hydrograph depicting Everglades Depth Estimation Network (EDEN) mean water depths (cm) during 1993–1999 and 2000–2013.

Models predicting species‐specific nesting effort included a subset of variables describing early dry season means, SEs, and monthly changes and means (January, February, and March) and were analyzed using a generalized linear mixed model (GLMM) in SAS 9.2 (SAS Institute 2010a). The random effect *Decade* was included in the models to account for the much wetter hydrologic regime that occurred in the study period from 1993 to 1999 (Fig. 4) with the expectation that species that rely more on concentrated prey would be negatively affected. Models predicting species‐specific nesting success included variables describing late dry season means, SEs, and monthly changes and means (February, March, and April) and were analyzed using a generalized linear model (GLM) in SAS 9.2 (SAS Institute 2010b). *A Priori* models were constructed (based on the historical and current timing of breeding responses; *n* = 27) and evaluated using Akaike's information criterion for small sample sizes (AIC_c_) to determine which models were most parsimonious (Burnham and Anderson 2002). Delta AIC [Δ_i_]) and AIC weights (*ω*_i_) were calculated from AIC_c_ values. Models with the lowest AIC_c_ value were considered the best explanatory models, although additional competing models with ΔAIC_c_ < 2 were considered equally plausible given the data (Burnham and Anderson 2002). Models with ΔAIC_c_ > 4 were considered to have little to no support (Burnham and Anderson 2002). Model‐averaged coefficients and standard errors (SE) were calculated for each parameter by averaging all models containing the variable in proportion to the *ω*_i_. The importance of a specific variable was determined by summing the weights of all models containing that term.

## Results

### Great Egret

**Figure 5:**
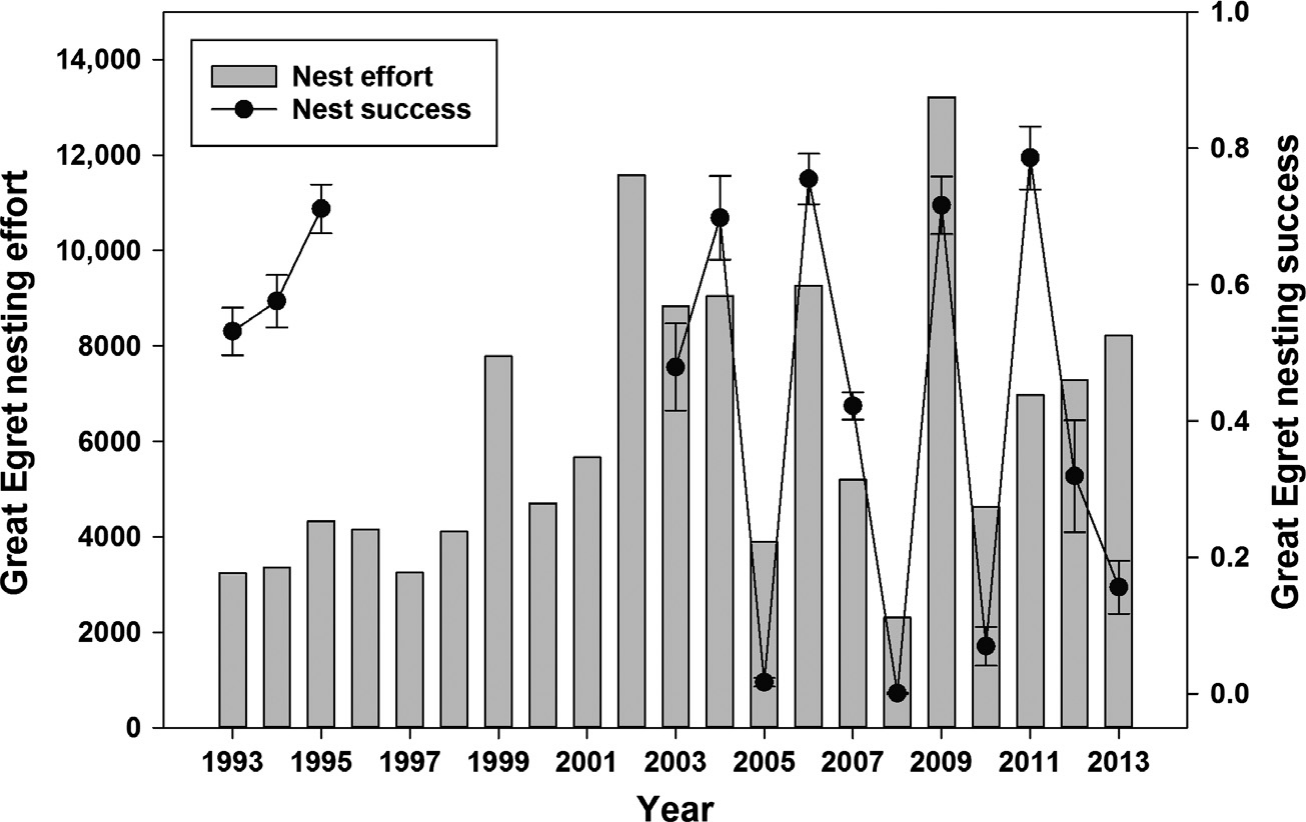
Great Egret nesting effort (i.e., max nesting pairs) and success (±SD) estimates from 1993 to 2013.

Egret nesting effort was moderately correlated with nesting success (*r*_s_ = 0.49, *P* = 0.07, *N* = 14) because there were years with low nest effort, but high success (e.g., 1993–1995) and years with high nest effort, but low success (e.g., 2012–2013; Fig. 5). The lowest egret nesting effort of 2308 nesting pairs was observed in 2008, and the highest nesting effort of 13,211 nesting pairs occurred in 2009. These two years also corresponded to the highest (2009: 0.72 ± 0.04) and lowest (2008: 0.00 ± 0.00) years of nesting success (Fig. 5). Year 2008 was characterized by low initial water levels and extreme reversals in the drying pattern during the breeding period, whereas initial water levels in 2009 were high and a steady water‐level recession was maintained throughout the breeding period.

Nesting effort of egrets was best explained by the model that included the variables for variability in the flocking ratio (*Ratio SE*), the mean change in March individual abundance (*Mar* Δ *TFC*), and the variability in the FI (*FI SE*; Table 1). The random effect *Decade* caused estimate of the variance component to be zero and thus was unrelated to variation in nesting effort after accounting for the fixed effects and was removed. Egret nesting effort was positively related to increased variability of the flocking ratio, caused by steady clustering of individuals into a smaller number of larger flocks. An increasing number of individuals in March (*Mar* Δ *TFC*), but steady FI was also related to higher nesting effort. The variable for change in individual abundance in March (*Mar* Δ *TFC*) received less support (0.52) than the variability in the FI (0.79) and variability in the flocking ratio (0.95), and the top model explained 61% of the variation in egret nesting effort (Table 1).

**Table 1:** Ranking of candidate models describing variables (temporal foraging conditions [TFC], foraging index [FI], and flocking ratio [Ratio]) influencing nesting effort (i.e., max nesting pairs) of Great Egrets, White Ibises, and Wood Storks in the Florida Everglades (Proc Mixed)

Great Egret nesting effort	*K* ^b^	AIC_c_^c^	Modelid	ΔAIC_c_^d^	*w*_i_^e^	*R* ^2^
Ratio SE, March Δ, FI SE	5	388.2	24	0.00	0.41	0.61
Ratio SE, April TFC, FI SE	5	391.0	9	2.76	0.10	
Ratio SE, March Δ	4	391.0	11	2.78	0.10	
Variable	*N*	Avg PE	SE	Importance		
Intercept	27	2716.707	2208.99	1.00		
Ratio SE	12	128,966.758	28,327.16	0.95		
FI SE	8	−0.547	0.26	0.79		
March Δ	9	13.892	9.68	0.52		
White Ibis nesting effort	*K* ^b^	AIC_c_^*c*^	Modelid	ΔAIC_c_^d^	*w*_i_^e^	*R* ^2^
Mean TFC, March Δ	5	445.2	7	0.00	0.61	0.76
March TFC, March Δ	6	448.5	10	3.36	0.11	
Variable	*N*	Average PE	SE	Importance		
Intercept	27	−9736.618	13,127.94	1.00		
March Δ	8	29.225	12.62	0.82		
Mean TFC	9	2.425	1.01	0.78		
Wood Stork nesting effort	*K* ^*b*^	AIC_c_^c^	Modelid	ΔAIC_c_^d^	*w*_i_^e^	*R* ^2^
March FI	4	65.2	22	0.00	0.74	0.73
February FI, March FI	5	68.7	8	3.41	0.13	
Variable	*N*	Average PE	SE	Importance		
Intercept	27	1.263	1.21	1.00		
March FI	9	0.003	0.00	0.98		

Models are ranked by differences in Akaike's information criterion, and only candidate models within ΔAIC_*c*_d ≤ 4.0 are presented. Model selection results are followed by model averaging results for each species. The *R* ^2^ represents the model fit for the estimated annual nesting effort versus model‐averaged predicted values.

The most parsimonious model to explain the nesting success of egrets included the terms for variability in the flocking ratio (*Ratio SE*), abundance of individuals in April (*Apr* $\bar{x}$ *TFC*), and variability in the FI (Table 2). Similar to nesting effort, egret nesting success increased with the variability of the flocking ratio through clustering of foraging individuals. Nesting success also increased with high individual abundance in April (*Apr* $\bar{x}$ *TFC*), and a steady FI. The sums of the variable weights indicate high variable importance for the flocking ratio (0.97) and moderate importance for individual abundance in April (0.59) and the variability in the FI SE (0.50), and the top model explained 75% of the variation in egret nesting success (Table 2).

**Table 2:** Ranking of candidate models describing variables (temporal foraging conditions [TFC], foraging index [FI], and flocking ratio [ratio]) influencing nesting success (Mayfield method) of Great Egrets and White Ibises in the Florida Everglades (Proc GLM)

Great Egret nesting success	*K* ^b^	AIC_c_^c^	Modelid	ΔAIC_c_^d^	*w*_i_^e^	*R* ^2^
Ratio SE, April TFC, FI SE	5	3.8	27	0.00	0.28	0.75
Ratio SE, April TFC	4	4.3	9	0.41	0.22	
Ratio SE, mean TFC, FI SE	5	4.4	4	0.46	0.22	
Variable	*N*	Average PE	SE	Importance		
Intercept	27	−0.716	0.35	1.00		
Ratio SE	12	10.145	2.66	0.97		
April TFC	8	0.000	0.00	0.59		
FI SE	8	−0.000	0.00	0.50		
White Ibis nesting success	*K* ^b^	AIC_c_^c^	Modelid	ΔAIC_c_^d^	*w*_i_^e^	*R* ^2^
April TFC	3	−12.9	27	0.00	0.78	0.80
April TFC, March Δ	4	−9.0	23	3.84	0.11	
Variable	*N*	Average PE	SE	Importance		
Intercept	27	−0.409	0.12	1.00		
April TFC	8	0.000	0.00	1.00		

Models are ranked by differences in Akaike's information criterion, and only candidate models within ΔAIC_c_ *d* ≤ 4.0 are presented. Model selection results are followed by model averaging results for each species. The *R* ^2^ represents the model fit for estimated annual nesting effort vs. model‐averaged predicted values.

### White ibis

**Figure 6:**
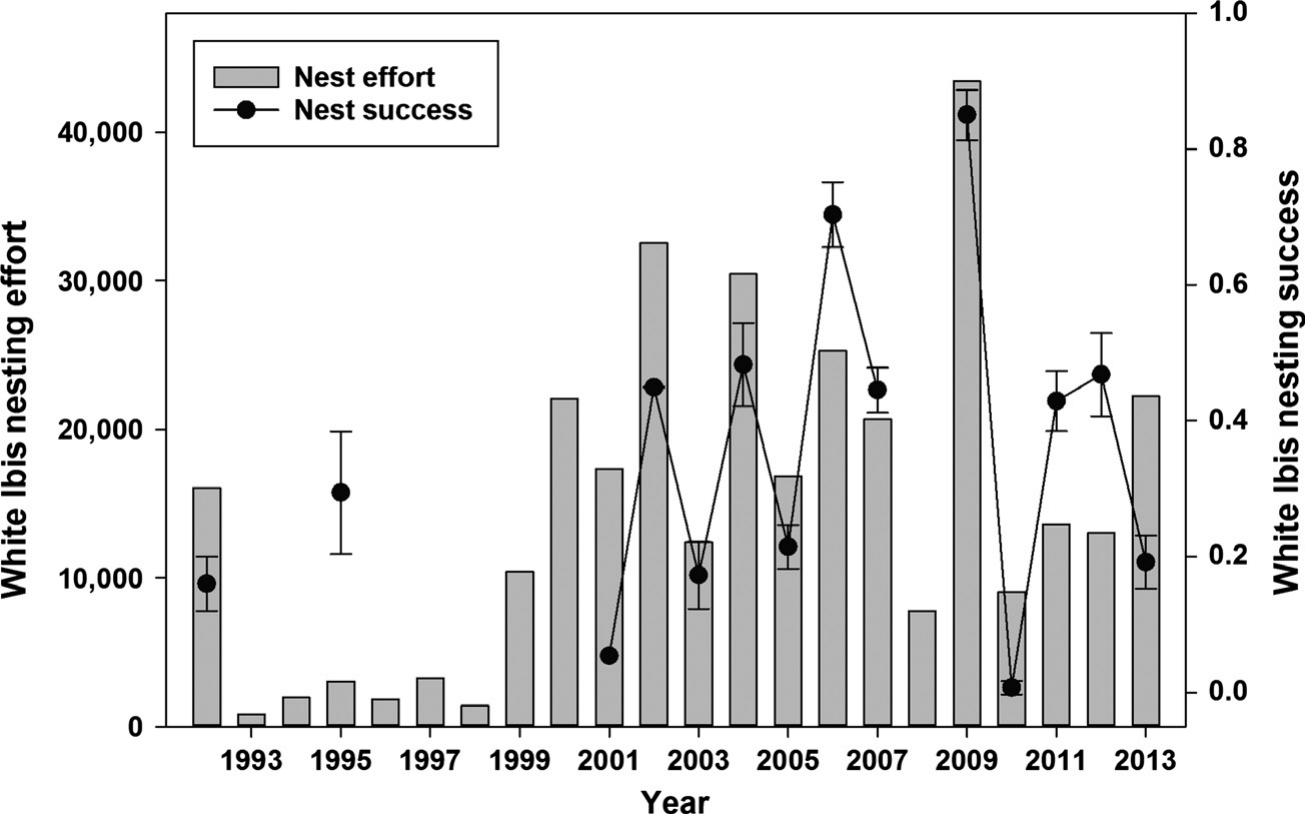
White Ibis nesting effort (i.e., max nesting pairs) and success (±SD) estimates from 1993 to 2013.

Ibis nesting success was more closely linked with nesting effort (*r*_s_ = 0.64, *P* = 0.02, *N* = 13), but similar to the egret, ibises had the highest nesting effort (43,415 nesting pairs) and success (0.85 ± 0.4) in 2009 (Fig. 6). Nesting effort was lowest in 1993 (818 nesting pairs), and nesting success was lowest in 2010 (0.01 ± 0.01), with both years having minimal water‐level recession.

The model that best described ibis nesting effort contained the terms describing mean abundance of individuals (*Mean TFC*), the change in March abundance (*Mar* Δ *TFC*), and *Decade* (Table 1). An overall negative effect on nesting effort of ibis was evident in the wetter period from 1993 to 1999 in comparison with 2000 to 2013. Ibis nests increased with increasing March and mean abundance, and variable importance was high for both of these (>0.7). The top model explained 76% of the variance in nesting effort (Table 1).

The top model for ibis nesting success included the term for high individual abundance in April (*Apr* $\bar{x}$ *TFC*; Table 2). Ibis nesting success increased with higher numbers of predicted individuals (*Apr* $\bar{x}$ *TFC*), which had a high variable importance (1.0), and this one term explained 80% of the variance in ibis nesting success (Table 2).

### Wood stork

**Figure 7:**
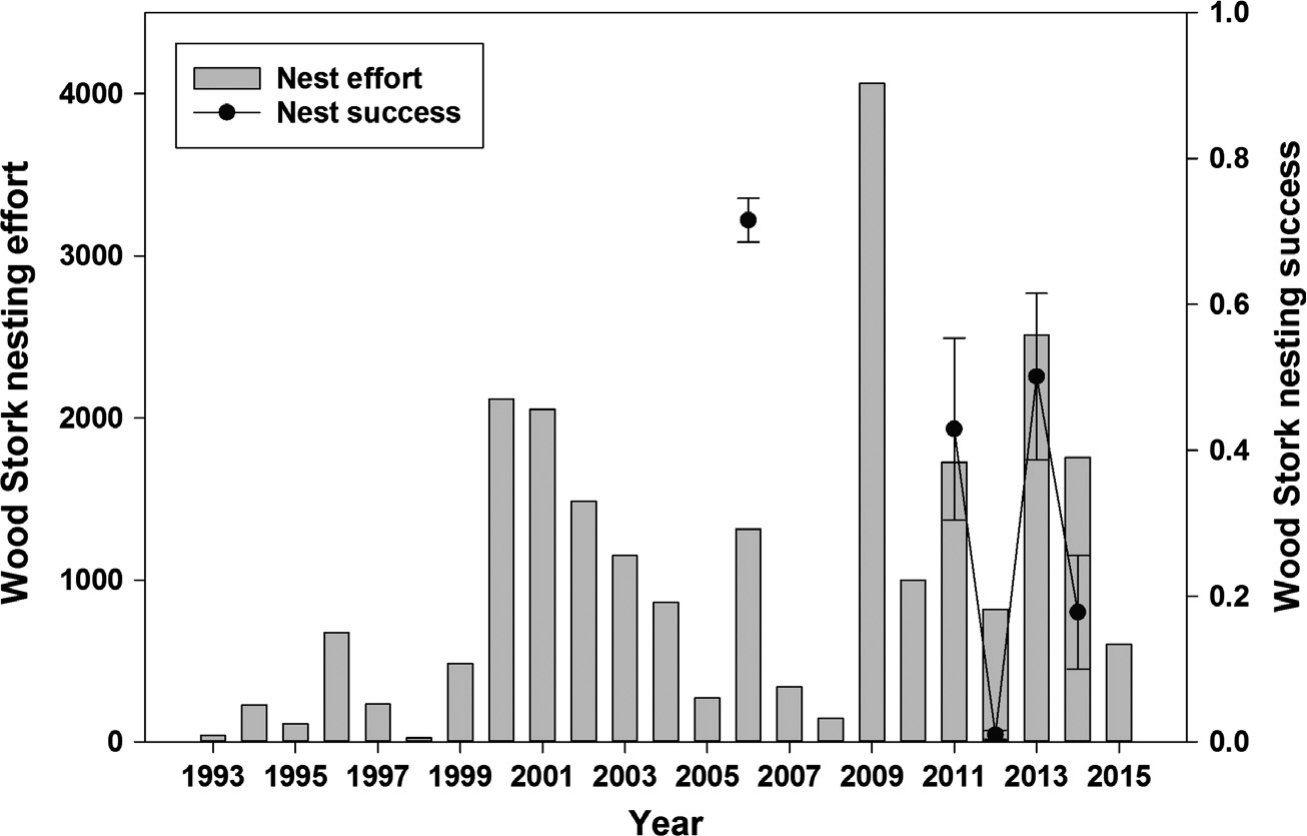
Wood Stork nesting effort (i.e., max nesting pairs) and success (±SD) estimates from 1993 to 2013.

Similar to the other species, stork nesting effort was highest in 2009 with 4063 nesting pairs, but the low of 25 nesting pairs occurred in 1998 (Fig. 7). The most parsimonious model to describe stork nesting effort consisted of the terms describing mean March FI and Decade (Table 1). Similar to ibises, an overall negative effect of high water conditions was evident from 1993 to 1999 on stork nesting effort, in comparison with the more recent hydrologic regime. Stork nests increased with a high March FI, when a peak in patch abundance (SFC) co‐occurred with high individual abundance (TFC).

## Discussion

This study provides the first empirical modeling framework that links daily changes in resource availability, habitat selection, and spatio‐temporal distributions (i.e., WADEM) to wading bird reproductive responses over periods of decades. The process used here allows managers to develop conservation strategies that (1) consider flexible behavioral patterns and (2) are robust to environmental variation over time. This work also showed that predictor variables at a variety of temporal resolutions (daily‐multiannual) and spatial scales (patch [400‐m] and regional) effectively explained variation in ecological processes that change habitat quality (McPherson and Jetz 2007), stressing the importance of a multiscaled species distribution modeling approach (Johnson et al. 2004, Flesch and Steidl 2010).

Because wading birds have flexible breeding schedules (Heath et al. 2003) and are often primarily limited by food availability (Ogden 1994, Gawlik 2002, Herring et al. 2011), it is not surprising that nesting responses were linked to the timing of increases in predicted individual bird and patch abundance. However, the fact that nesting effort is highly variable (CV = 70.4) suggests either that large sections of the population are not breeding in many years, or that annual breeding strategies by these highly mobile animals are formed by choices made at larger range‐wide scales (Frederick et al. 1996, Frederick and Ogden 1997).

The timing and overall magnitude of resource pulses indicated by WADEM outputs for habitat selection and individual species abundance were strongly linked to both ibis nesting effort and nesting success. Increases in abundance in March followed by sustained abundance in April strongly predicted ibis nesting effort and success, respectively. Overall high abundance from January to May explained an additional increase in nesting effort, with the earlier period likely driving the initiation response. Because most chicks are fledged in May, it is expected that high abundance in May is also related to fledging success and survival (Frederick and Spalding 1994). Therefore, increasing species abundance in March and sustained abundance in April–May would indicate successful ibis reproduction in their relatively short nesting cycle of 60–80 days (Frederick et al. 2009). Ibises also provided a contrast in wetland function between very wet conditions (middle 1990s) when ibis nesting effort and success was markedly low, and later, drier years when nesting effort and success improved markedly through increases in the availability of prey. Indeed, the historical benchmark of a 1.6‐year interval between exceptional ibis nesting events (>16,977 nesting pairs; Frederick et al. 2009) has been achieved over the last 5 years (Frederick 2013).

Similar to ibises, nesting effort and success of egrets increased with increasing TFC model predictions for species abundance in March and high predictions for April, respectively. However, variable importance was lower for those time‐sensitive estimates than for metrics that described foraging conditions over the whole breeding season. In particular, egret nesting effort and success were higher in years when a high abundance of foraging egrets clustered into fewer flocks. Great Egrets forage solitarily more than do ibises, because the stalking, visual foraging of egrets is more sensitive to interference by other individuals (Gawlik 2002). The relationship we found between flocking ratio and nesting effort and success therefore suggests that an increase in conspecific attraction for species with high interference costs can be used as a measure to describe increasing habitat quality (Folmer and Piersma 2012). Further, increased social attraction under improving foraging conditions could account for a decline in the predictive power (area under the curve; AUC) of resource selection functions for egrets using solely environmental variables (Beerens et al. 2011).

After the above fixed effects were accounted for, we found that egret nesting effort was not affected by the wetter conditions of the 1990s and nesting success was high from 1993 to 1995. By comparison with ibises, these wetter conditions still favored egrets because of their longer legs and stalking foraging habit, allowing a broader depth tolerance (Gawlik 2002, Beerens et al. 2011) and preference to exploit larger prey that develop over a longer period of inundation (up to 600 days; Beerens et al. 2015). On a much larger time scale, these features are borne out in a decades‐long increase of breeding egrets (a visual forager) relative to tactile foragers (ibises and storks), suggesting habitat quality has generally declined in the Everglades for species with more specialized habitat requirements that rely on higher prey concentrations (Ogden 1994, Gawlik 2002, Beerens et al. 2011).

For both egrets and ibises, breeding responses were more closely linked with individual species abundance (TFC model outputs) than with patch abundance (SFC model outputs). However, the TFC models also showed that species abundance increased when landscape depth heterogeneity (SD) was high and individuals had a variety of depth choices within a region. For egrets, a stable FI incorporating patch abundance was related to increases in nesting effort and success. Because the Everglades landscape slopes only minutely (3–6 cm/km; Givnish et al. 2008), patch abundance is expected to be low early in the wet season when most of the wetland is too deep to forage. As the dry season progresses and a larger portion of the landscape is within suitable depths, patch abundance peaks, then eventually declines as most of the landscape dries. The egret FI remained stable as long as predicted species abundance was high, even if patch abundance declined. Abundance remained high during drier conditions (i.e., shallow depths) only when sites had not dried within a year (e.g., long DSD), again demonstrating the contrast in processes that control food availability over long and short time scales.

Wood Stork nesting effort was highest when both individual and patch abundance were high. In contrast to egret and ibis nesting effort, which responded mostly to increasing abundance in March, stork nesting effort was high only when both individual and patch abundance in March were high. Although we could not model stork nesting success, we hypothesize that a sustained high FI is crucial to reproductive success, partly because of the very high energetic needs of this species when feeding nestlings, and partly because storks have a very long reproductive cycle (e.g., >110 days, Kahl 1964). Indeed, many years with high nesting success in storks also had a high FI in April and May, whereas low scores in April and May were associated with increased nest abandonment (Beerens 2014).

### Application

Output from hydrologic models simulating restoration alternatives can be used as input for WADEM to evaluate the response to restoration alternatives by wading birds. There is also much impetus to discover restoration scenarios that produce high estimates of nest success in years with predictions of high nesting effort (i.e., nesting effort × nesting success). Additionally, WADEM is utilized to inform water management operations in real time as well as to predict the effects of longer‐term stressors like climate change on wading bird habitat quality (Catano et al. 2015).

In dynamic environments, species distribution responses to resources are often noisy and can be difficult to identify, but incorporating components of species ecology such as flexible selection of resources at several temporal scales, responses to environmental gradients, conspecific attraction, and spatial autocorrelation can yield results that better approximate habitat quality. Furthermore, responses to changes in timing of habitat quality at different phases of breeding can provide inferences to population‐level changes that may result from restoration and/or climate change.

## Conflict of Interest

None declared.
